# Supplementary material for: Cancer Survivors in Saint Lucia Deeply Value Social Support: Considerations for Cancer Control in Under-Resourced Communities
Source: Int J Environ Res Public Health. 2022 May 27;19(11):6531. doi: 10.3390/ijerph19116531 (PMC9180699; doi:10.3390/ijerph19116531)
Supplement: Supplementary file 1 [file ijerph-19-06531-s001.zip › ijerph-1696392-supplementary.pdf]

Title: Cancer survivors in Saint Lucia deeply value social support: considerations for cancer control in under-resourced communities

Aviane Auguste, Shania Cox, JoAnn S Oliver, Dorothy Phillip, Owen Gabriel, James St.Catherine, Carlene Radix, Danièle Luce and Christine Barul

Supplementary materials

Table S1: Characteristics of cancer patients who were represented by a caregiver during the study interview.

Variable	Patient n°		
	17	18	19
Sex	Female	Female	Male
Age at diagnosis (y)	69	55	60
Cancer site	Endometrium	Breast	Colon
Survivorship (y)	<1	<1	<1
Stage at diagnosis	Missing	IV	IV
Vital status at interview	Alive	Dead	Alive
Treatment status	No treatment	No treatment	Still on-treatment
Marital status	Married/Other	Married/Other	Single
Education level	Primary	Secondary	Primary
Private medical insurance	No	No	No
Hot water at home	No	No	No
History of medical condition(s)	No	No	Yes
Professional status	Not working	Not working	Missing
Treatment abroad	NA	NA	No
Diagnostic test(s) abroad	Missing	Yes	No

NA: Not applicable

Table S2: Caregiver responses for the three open-ended questions from the interview.

Open-ended question	Caregiver n°	Quotes
Was there anything in particular that made your experience easier?	*17*	*NA*
	18	*She (the patient) has a family member who is able to send her for a different opinion on her illness*
	19	*NA*
Was there anything in particular that made your experience harder?	*17*	*Doctors might have realised it was cancer and never told me. Doctors need to be professional at all times regardless of how difficult it is for persons who give care or patients themselves*
	18	*Family not knowing she (the patient) is sick*
	19	*There were many hitches in the care of my brother during his illness but the one which stands out to me is the delay in the follow up with the oncologist because of the change of oncologist.*
Do you have any suggestions to help improve the experience for other people in similar circumstances?	*17*	*Seek help from persons with similar illnesses. Go to your health care provider if unexplained changes occur in your body. Do not keep your diagnosis to yourself as it might be more difficult to deal with.*
	18	*Tell loved one how you feel. Do not be ashamed to discuss/disclose body changes with loved ones. Do your annual check-up.*
	19	*I believe that there should be more than one specialist available in the event that one is absent. Patients will not be left stranded and eventually die*

NA: No response provided for this question
